# Anomalous Right Coronary Artery Intervention Guided by Patient-Specific 3-Dimensional Printed Modeling

**DOI:** 10.1016/j.jaccas.2026.107757

**Published:** 2026-04-02

**Authors:** Adrien Jossart, Timothée Noterdaeme, Olivier Gach, Giuseppe Colletti, Marouane Boukhris, Claudiu Ungureanu

**Affiliations:** a Department of Cardiology, Hôpital de Jolimont, La Louvière, Belgium; b Department of Cardiology, CHC Mont-Légia, Liège, Belgium; c Department of Cardiology, Clinique Saint Joseph, Vivalia, Arlon, Belgium; d Department of Cardiology, CHU Saint-Etienne, Saint-Etienne, France

**Keywords:** 3D printing, guiding catheter, anomalous aortic origin of a coronary artery, percutaneous coronary intervention

## Abstract

**Background:**

Percutaneous coronary intervention (PCI) of an anomalous aortic origin of a coronary artery (AAOCA) is technically challenging given difficult engagement and poor support. While coronary computed tomography angiography aids planning, it lacks the tactile simulation provided by patient-specific 3-dimensional (3D) modeling.

**Case Summary:**

A 58-year-old woman presented with recurrent angina and an anomalous right coronary artery originating from the left sinus. After repeated failures using conventional catheter selection, a 1:1 scale 3D-printed model was used for bench testing. This enabled the selection of an EBU 3.0 guiding catheter, leading to a successful PCI with 2 drug-eluting stents.

**Discussion:**

This case demonstrates that 3D bench testing allows operators to anticipate support limitations and select optimal catheter geometry, potentially reducing procedure time and radiation. It highlights a structured alternative to empirical “trial-and-error” strategies in complex congenital anatomy.

**Take-Home Message:**

Patient-specific 3D modeling facilitates successful PCI in AAOCA by enabling precise preprocedural simulation and optimal guiding catheter selection.

## Visual Summary

**Figure:**
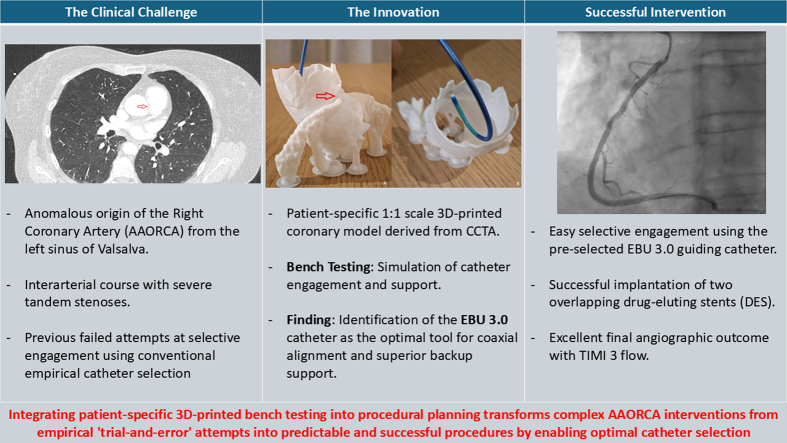

**Visual Summary:**
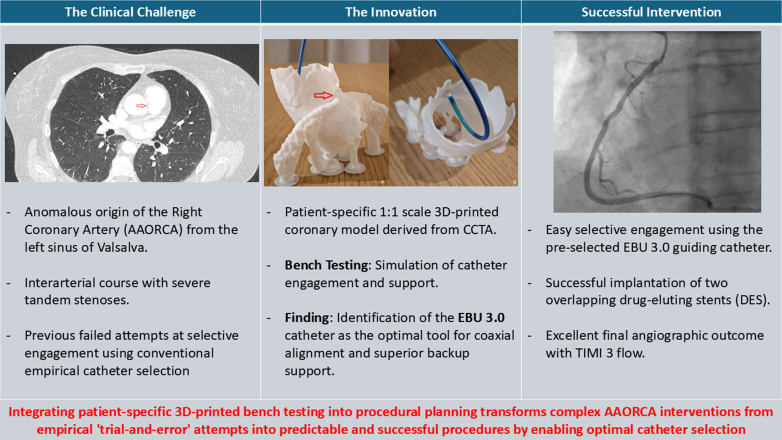
3D-Guided PCI in Complex AAORCA (Left) The Clinical Challenge: Symptomatic anomalous right coronary artery with failed conventional engagement attempts. (Middle) The Innovation: Patient-specific 1:1 scale 3D-printed model derived from CCTA for preprocedural bench testing. (Right) Successful Intervention: Identification of EBU 3.0 as the optimal catheter, leading to successful revascularization with drug-eluting stents and TIMI flow grade 3. Conclusion: 3D-printed bench testing transforms empirical “trial-and-error” into a predictable and successful procedural strategy. 3D = 3-dimensional; AAORCA = anomalous aortic origin of the right coronary artery; CCTA = coronary computed tomography angiography; PCI = percutaneous coronary intervention.

Anomalous aortic origin of a coronary artery (AAOCA) is a rare congenital condition, with a reported prevalence ranging from 0.1% to 1% in the general population,^1^ and with anomalous aortic origin of the right coronary artery (AAORCA) representing the most frequent variant.^2^ Although uncommon, AAOCA may be clinically relevant, particularly when associated with myocardial ischemia or acute coronary syndromes.^1^

**Take-Home Messages**

• Patient-specific 3D-printed coronary modeling facilitates successful PCI in complex AAORCA by allowing for physical bench testing and precise guiding catheter selection, effectively overcoming the limitations of conventional empirical strategies.
• Integrating 3D simulation into preprocedural planning transforms static imaging into a dynamic technical rehearsal, which can optimize procedural efficiency and reduce radiation and contrast exposure in patients with challenging congenital coronary anatomy.

The role of percutaneous coronary intervention (PCI) in the management of AAOCA has progressively emerged over the past decade. The 2017 American Association for Thoracic Surgery guidelines were the first to acknowledge PCI as a potential therapeutic option for AAOCA in selected patients (Class IIb, Level of Evidence: C),^1^ after case series demonstrated technical feasibility.^3^ More recently, PCI has been increasingly considered in patients older than 30 years with AAORCA and clinical symptoms suggestive of ischemia or objectively documented myocardial ischemia, especially in the presence of atherosclerotic coronary disease.^4^

However, PCI in the setting of anomalous coronary anatomy is often technically challenging. Atypical ostial location, acute takeoff angulation, and an unusual proximal vessel course may render standard diagnostic and guiding catheter shapes inadequate, leading to difficult selective engagement, poor coaxiality, insufficient guide support, and prolonged procedures.^5^ These challenges are particularly critical in acute coronary syndromes, where rapid vessel engagement, procedural efficiency, and minimization of contrast and radiation exposure are essential.

Preprocedural coronary computed tomography angiography (CCTA) has been shown to improve anatomical understanding and procedural planning in coronary interventions.^6^ Nevertheless, most supporting data derive from patients with normally originating coronary arteries. More recently, patient-specific, full-scale (1:1) 3-dimensional (3D) coronary models have been proposed as an additional planning tool in complex anatomy, allowing operators to simulate catheter engagement, test different guiding configurations, and anticipate support limitations prior to the procedure.^7^

We report a clinical case in which successful PCI of an AAORCA was achieved after personalized 3D procedural planning, after repeated failure of conventional catheter strategies.

## Case Presentation

A 58-year-old woman with no known history of coronary artery disease and an active smoking habit presented with acute retrosternal chest pain associated with posterior ST-segment elevation on electrocardiogram. Emergency coronary angiography demonstrated an acute thrombotic occlusion of the obtuse marginal branch. Primary PCI was performed with deployment of 2 overlapping drug-eluting stents (DESs) (2.75 × 20 mm and 2.75 × 15 mm), achieving an optimal angiographic result. Proximal optimization technique was performed using a 3.00-mm noncompliant balloon, followed by final kissing balloon inflation with 3.00-mm and 2.75-mm balloons. A severe proximal left anterior descending artery (LAD) stenosis was also identified, and staged PCI was planned. The right coronary artery (RCA) could not be visualized despite multiple angiographic projections, raising suspicion of an anomalous origin, and CCTA was therefore recommended.

The patient had a favorable clinical course, with preserved left ventricular systolic function on transthoracic echocardiography, except for basal and mid inferoseptal hypokinesia. She was discharged on guideline-directed medical therapy.

Two weeks later, before undergoing the planned CCTA, the patient was readmitted with recurrent typical anginal chest pain. Staged PCI of the proximal LAD was performed with implantation of a 3.0 × 48 mm DES, followed by proximal optimalization technique using a 3.5-mm noncompliant balloon and final kissing balloon inflation toward the diagonal branch, with an excellent angiographic result. During the same procedure, a second operator attempted to engage the right coronary ostium using multiple guiding catheter shapes and types via femoral access; however, selective engagement of the RCA remained unsuccessful.

**Figure 1:**
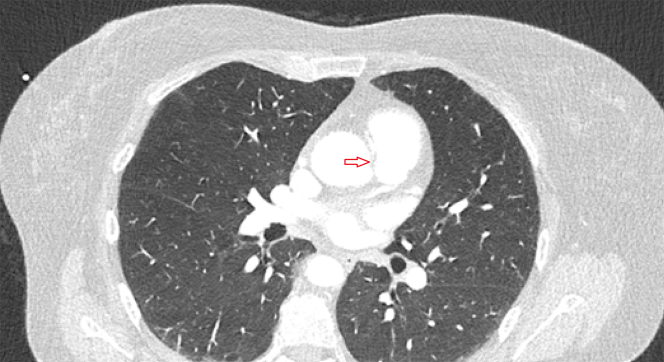
Preprocedural Cardiac Computed Tomography Angiography Axial view of the CCTA demonstrating the AAORCA (red arrow). The vessel originates from the left coronary sinus and follows an interarterial course between the aorta and the pulmonary artery. AAORCA = anomalous aortic origin of the right coronary artery; CCTA = coronary computed tomography angiography.

Subsequent CCTA revealed an anomalous RCA originating from the left coronary sinus and following an interarterial course (Figure 1), with severe stenoses in its mid and distal segments. To determine the most appropriate guiding catheter and engagement strategy, a patient-specific 3D-printed coronary model was generated from the computed tomography dataset. A 3D reconstruction was created using an open-source DICOM (Digital Imaging and Communications in Medicine) viewer via targeted segmentation. The models were exported in standard tessellation language format and postprocessed in Blender to correct mesh errors and apply a “solidify” modifier. The finalized mesh was printed using a high-resolution fused deposition modeling 3D printer with polylactic acid filament. Notably, the total processing time was under 30 minutes, followed by a 1-hour print duration.

**Figure 2:**
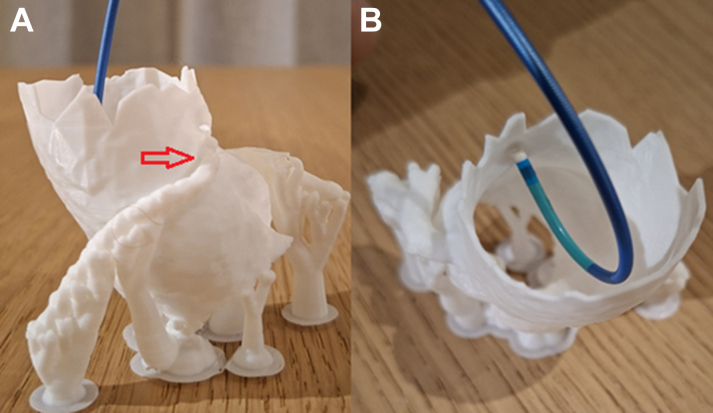
Preprocedural Engagement and Guiding Catheter Strategy on a 3D-Printed Coronary Model (A) Engagement of the AAORCA (red arrow) on a patient-specific 3D printed coronary model using a 6-F EBU 3.0 guiding catheter. (B) The EBU 3.0 guiding catheter demonstrates excellent coaxial alignment with the coronary ostium and provides sufficient guide support. AAORCA = anomalous aortic origin of the right coronary artery.

After multiple bench-testing simulations, 2 guiding catheters—the RCB (right coronary bypass) catheter and the EBU (extra back-up) 3.0 catheter—were able to engage the RCA ostium; however, the EBU 3.0 catheter provided superior support (Figure 2).

**Figure 3:**
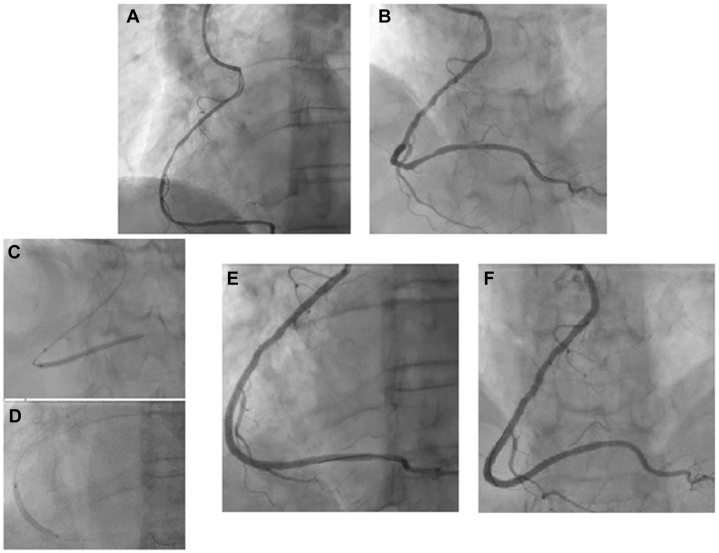
Stepwise PCI Strategy for Anomalous RCA Using 3D-Guided Catheter Selection (A and B) Selective engagement of the anomalous RCA using an EBU 3.0 guiding catheter. (C and D) Delivery of balloons and overlapping stents using EBU 3.0 guiding catheter with 5.5-F GuideLiner. (E and F) Final angiographic result with TIMI flow grade 3. 3D = 3-dimensional; PCI = percutaneous coronary intervention; RCA = right coronary artery.

After bench testing, the procedure was scheduled, and the operator was able to easily engage the RCA using the EBU 3.0 guiding catheter. A 5.5-F guide extension catheter was used to further enhance guide support. Two overlapping DESs (2.5 × 48 mm and 2.75 × 33 mm) were successfully implanted, resulting in an excellent angiographic outcome, with final TIMI flow grade 3 (Figure 3). Intravascular imaging was not performed in this case because of the challenging coronary engagement and the need to preserve maximal guide stability and support.

The patient was discharged on dual antiplatelet therapy without complications. At the 3-month clinical follow-up, she remained free of angina and reported no limitations in daily activities.

## Discussion

The present clinical report highlights the value of comprehensive preprocedural planning that combines CCTA with patient-specific 3D bench testing prior to attempting PCI in a patient with AAORCA. CCTA plays a pivotal role in the assessment of complex coronary anatomy by providing high-resolution 3D visualization of the coronary origin, ostial morphology, spatial orientation within the aortic root, and proximal vessel course.

Importantly, the clinical utility of CCTA-guided planning is not limited to congenital coronary anomalies. In routine interventional practice, CCTA-based planning has been shown to meaningfully influence procedural strategy by enabling detailed assessment of coronary origin, plaque distribution, calcification burden, and lesion geometry, thereby allowing anticipatory procedural adaptation.^6^ In the setting of chronic total occlusion interventions, both randomized and observational studies have demonstrated higher technical success rates with CCTA guidance compared with angiography alone (approximately 90%-94% vs 84%-88%).^8^ In a pilot randomized study focusing on aorto-ostial right coronary lesions, preprocedural 3D computed tomography planning was associated with an approximately 50% reduction in contrast volume (≈60 vs 115 mL), a significant reduction in radiation dose (≈210 vs 500 mGy), and shorter procedure duration (≈30 vs 50 minutes) compared with angiography-guided PCI.^9^

The addition of patient-specific 3D bench testing using a printed coronary model further strengthens this planning paradigm. Bench testing enables direct evaluation of guiding catheter behavior within a realistic anatomical replica, including the ability to achieve coaxial ostial engagement and provide sufficient support for device delivery. Beyond technical assessment, this approach enhances the operator's understanding of the individual coronary anatomy and facilitates mental rehearsal of catheter-aorta-coronary interactions, which may translate into improved intraprocedural performance. Previous studies have suggested that 3D bench testing assists operators in determining optimal guiding catheter geometry, improves procedural predictability, and reduces trial-and-error catheterization attempts.^7^

The procedural complexity associated with AAORCA is illustrated by a previously reported series of PCI performed without dedicated 3D planning,^10^ in which mean fluoroscopy time reached 20.7 minutes (range: 12.2-63.3 minutes) and mean contrast volume was 210 mL (range: 120-320 mL), reflecting repeated catheter manipulations and engagement difficulties. In the present case, complete revascularization was considered clinically important; however, despite 2 attempts by experienced operators using different vascular access sites (radial and femoral) and multiple guiding catheter shapes, selective engagement of the right coronary ostium could not be achieved, underscoring the limitations of conventional empirical catheter selection in this anatomical setting.

In contrast, personalized bench testing using a patient-specific 3D coronary model provided a structured and reproducible alternative to conventional planning. Although 3D printing and bench testing require specialized technical expertise, our streamlined workflow—from DICOM import to the completed 1:1 model—required only 90 minutes. This rapid turnaround, combined with the use of low-cost open-source software and standard consumables, significantly reduces the traditional barriers of time and cost. While not suitable for emergency ad hoc PCI, this approach is highly scalable for elective complex cases where conventional strategies have failed. This strategy proved particularly valuable after failed initial attempts and may be especially useful when AAORCA is identified preprocedurally or suspected during diagnostic angiography. Rather than pursuing further empirical catheter manipulation, the combination of CCTA and 3D bench testing allows the procedural approach to be redesigned based on objective anatomical and mechanical insights, with the potential to improve procedural efficiency, reduce contrast and radiation exposure, and enhance procedural safety.

### Limitations

This report describes a single clinical case, and its findings cannot be generalized to all patients with anomalous coronary anatomy. Furthermore, 3D printing and bench testing require high-quality imaging, specialized technical expertise, and additional preparation time, which may limit their routine applicability, particularly in urgent or emergent clinical settings. Finally, although advanced preprocedural planning may theoretically reduce procedural inefficiency, radiation exposure, and contrast use, these parameters were not prospectively compared with a conventional strategy in the present case and therefore remain hypothesis-generating.

## Conclusions

Personalized procedural planning combining CCTA with patient-specific 3D coronary bench testing may substantially facilitate PCI in patients with anomalous coronary anatomy. By enabling optimal guiding catheter selection and procedural strategy definition before the intervention, this approach has the potential to improve procedural success while reducing procedure duration, radiation exposure, contrast volume, and unnecessary guiding catheter exchanges.

## Funding Support and Author Disclosures

The authors have reported that they have no relationships relevant to the contents of this paper to disclose.
